# A passive flow microreactor for urine creatinine test

**DOI:** 10.1038/s41378-025-00880-z

**Published:** 2025-04-02

**Authors:** Dumitru Tomsa, Yang Liu, Amanda Stefanson, Xiaoou Ren, AbdulRazaq A. H. Sokoro, Paul Komenda, Navdeep Tangri, Rene P. Zahedi, Claudio Rigatto, Francis Lin

**Affiliations:** 1 Department of Physics and Astronomy, University of Manitoba, Winnipeg, MB R3T 2N2 Canada; 2 Institute of Health Sciences and Technology, Institutes of Material Science and Information Technology, Anhui University, Hefei, 230601 China; 3 Department of Pathology, University of Manitoba, Winnipeg, MB R3P 3E5 Canada; 4 Department of Internal Medicine, University of Manitoba, Winnipeg, MB R3A 1R9 Canada; 5 Manitoba Centre for Proteomics and Systems Biology, Winnipeg, MB R3E 3P4 Canada

**Keywords:** Engineering, Chemistry

## Abstract

Chronic kidney disease (CKD) significantly affects people’s health and quality of life and presents a high economic burden worldwide. There are well-established biomarkers for CKD diagnosis. However, the existing routine standard tests are lab-based and governed by strict regulations. Creatinine is commonly measured as a filtration biomarker in blood to determine estimated Glomerular Filtration Rate (eGFR), as well as a normalization factor to calculate urinary Albumin-to-Creatinine Ratio (uACR) for CKD evaluation. In this study, we developed a passive flow microreactor for colorimetric urine creatinine measurement (uCR-Chip), which is highly amenable to integration with our previously developed microfluidic urine albumin assay. The combination of the 2-phase pressure compensation (2-PPC) technique and microfluidic channel network design accurately controls the fluidic mixing ratio and chemical reaction. Together with an optimized observation window (OW) design, a uniform and stable detection signal was achieved within 7 min. The color signal was measured by a simple USB microscope-based platform to quantify creatinine concentration in the sample. The combination of the custom in-house photomask production techniques and dry-film photoresist-based lithography enabled rapid iterative design optimization and precise chip fabrication. The developed assay achieved a dynamic linear detection range up to 40 mM and a lower limit of detection (LOD) of 0.521 mM, meeting the clinical precision requirements (comparable to existing point-of-care (PoC) systems). The microreactor was validated using creatinine standards spiked into commercial artificial urine that mimics physiological matrix. Our results showed acceptable recovery rate and low matrix effect, especially for the low creatinine concentration range in comparison to a commercial PoC uACR test. Altogether, the developed uCR-Chip offers a viable PoC test for CKD assessment and provides a potential platform technology to measure various disease biomarkers.

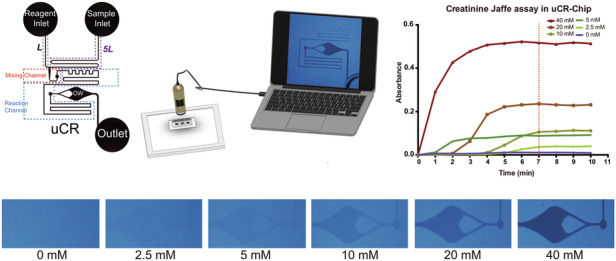

## Introduction

Chronic kidney disease (CKD) is characterized by kidney damage or loss of function that persists for at least 3 months^1^. Kidney function declines over time, causing fluid, electrolyte, and waste buildup in the body. According to a recent analysis, CKD is estimated to affect 843.6 million individuals around the world or 8–16% of the global population, making it a major cause of death, particularly in lower- and middle-income nations^2–5^. As highlighted by the Centers for Disease Control and Prevention guidelines, CKD is closely associated with heart disease, high blood pressure, diabetes, and cardiovascular-kidney-metabolic syndrome^6,7^. Additionally, recent data analysis of hospitalized COVID-19 patients revealed CKD as a risk factor for medical complications^8^.CKD can progress to an advanced stage and eventually requires treatments such as peritoneal dialysis, hemodialysis or kidney transplantation, which places a heavy burden on the healthcare system and significantly affects patients’ well-being^2^. CKD can be treated effectively and the consequence are preventable if diagnosed at an early stage^9^. However, fewer than 5% of those in the early stages of CKD are aware of their disease^10^. Effective screening strategies with at-risk patients could increase the CKD identification and is predicted to be cost-effective. The two key diagnostic markers for CKD are the estimated glomerular filtration rate (eGFR) and the Urine Albumin-to-Creatinine Ratio (uACR), both involving measuring creatinine as a biomarker. The standard CKD diagnosis is based on eGFR of less than 60 mL/min per 1.73 m^2^ of body surface area over at least 3 months, measured from a blood filtration marker such as serum creatinine^11^. In addition, uACR is an important measurement for identifying kidney damage in patients with kidney-related diseases. The urine tests are often the first step in CKD diagnosis and a critical part of follow-up management^12^. As a non-invasive assay, urine tests not only provide diagnostic and staging data for CKD, but also generate insights into prognostics and progression of the disease^13^. Furthermore, low levels of creatinine in urine can indicate low muscle mass, thus are considered a prognostic factor for predicting cardiovascular disease and CKD outcomes^14^.

Creatinine is a small molecule (0.113 kDa) by-product of creatine metabolism in muscle tissue. Muscle creatine is used to produce energy in the form of ATP via the enzymatic conversion to phosphocreatine. Creatinine is formed by spontaneous, non-enzymatic degradation of creatine, which is then released into the bloodstream and ultimately filtered by the kidneys as a urinary waste product^15^. As creatinine is secreted at a relatively constant rate compared to urine albumin, it is commonly used as a normalization factor in urinary analysis, particularly to determine uACR as more accurate CKD screening and diagnostic marker than urine albumin alone^11^.

Clinical urine tests are typically conducted in centralized labs, requiring patients to pay a separate lab visit, and the sample-to-result time is long^16^. A rapid, cost-effective, easy-to-use, and accurate point-of-care (PoC) urine test is highly desirable^17^. As such, dipstick strips are commonly used PoC urine test tools, which provide qualitative or semi-quantitative test results^17^. More accurate PoC urine tests are available; however, the current systems remain cost prohibitive and require specialized skills^18^. The powerful potential of microfluidic devices for PoC diagnostic applications lies in the miniaturization of chemical assays and the simplification of their use, which is the cornerstone of their demonstrated promise to fully address the well-accepted ASSURED criteria for PoC diagnostics^19^. Toward this direction, we previously developed a passive microfluidic device for quantitative urine albumin measurement at very low cost (uAL-Chip) and validated its effective use for testing clinical CKD samples^20^. Because of the importance of urine creatinine to determine uACR, a passive microfluidic device similar to the uAL-Chip for urine creatinine measurement is highly desirable. Therefore, in the current study, we further developed a passive flow microreactor for measuring urine creatinine (uCR-Chip) based on the Jaffe reaction^21,22^. The uCR-Chip integrates the oil coverage-based 2-phase pressure compensation (2-PPC) technique with optimized microfluidic channel designs to enable highly controlled fluidic mixing and chemical reaction leading to rapid stabilization of the colorimetric detection signal. Here, we present the details of the uCR-Chip design, fabrication, optimization, and test methodology, as well as its technical characterization and validation results.

## Results

### Controlled passive flow mixing and chemical reaction

The Jaffe reaction between creatinine and picric acid in an alkaline medium produces the Janovsky complex, which exhibits a distinct orange color by absorbing light in the wavelength range of 490 nm to 510 nm^22^. The low reagent cost and simplicity of this reaction make it an attractive colorimetric method for quantifying creatinine concentration in urine and blood. Therefore, the main requirements for the Jaffe microreactor are to completely mix the sample with the reagents at a defined ratio, control the reaction time, and allow for simple and reliable colorimetric signal readout, which guided the design, fabrication, and operation of the uCR-Chip.

Theoretically, the hydraulic resistance of the microfluidic channel is directly proportional to the channel length (Eq. 1 for microfluidic channels with rectangular cross section^23,24^).

1 $$R=\frac{12\mu L}{w{h}^{3}}{\left[1-\frac{h}{w}\left(\frac{192}{{\pi }^{5}}{\sum }_{n=\mathrm{1,3,5}}^{\infty }\frac{1}{{n}^{5}}\tanh \left(\frac{n\pi w}{2h}\right)\right)\right]}^{-1}$$

where *R* is the hydraulic resistance, *L* is the channel length, *w* is the channel width, *h* is the channel height and *μ* is viscosity of the fluid.

Therefore, the volumetric flow rate is inversely proportional to the channel resistance.

2 $$Q=\,\frac{{P}_{2}-{P}_{1}}{R}$$

Where *Q* is the flow rate; *P2-P1* is the pressure difference between the two ends of the channel.

Consequently, the mixing ratio between the sample and reagent can be determined by designing the inlet channels with the inverted length ratio^20^, which guides the inlet channels design.

**Fig. 1:**
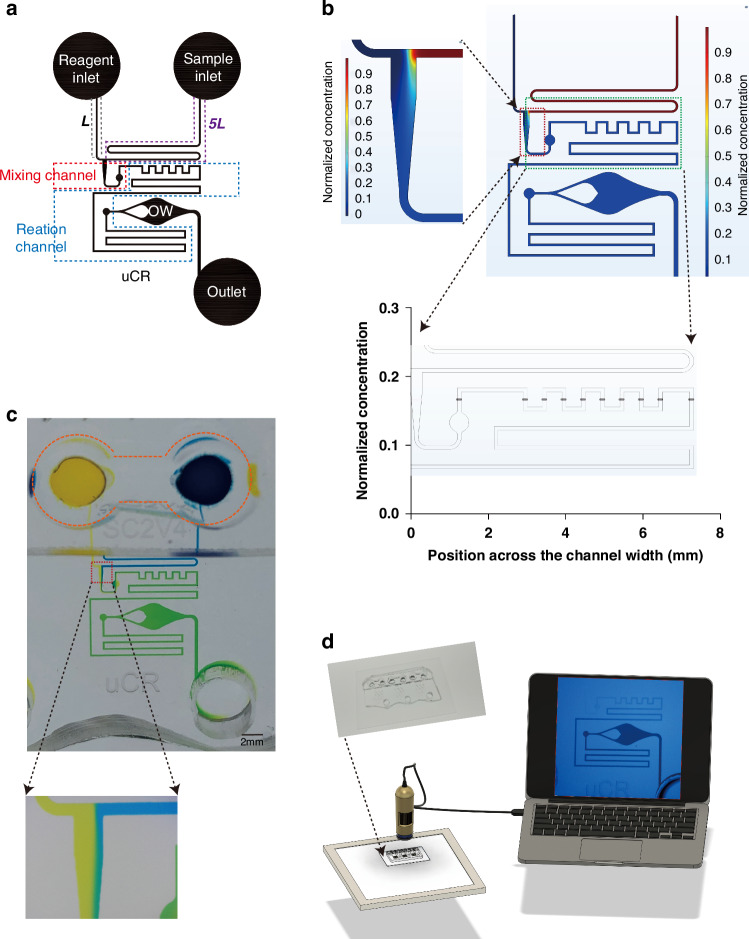
. **Illustration of the uCR-Chip and the test system**. **a** Schematic illustration of the uCR-Chip showing the reagent inlet, sample inlet, observation window (OW), and outlet. The notation L and 5L indicate the channel length from the reagent inlet and the sample inlet channel, respectively, with a ratio of 1:5. The mixing channel originates from the intersection of the two flows, followed by the reaction channel before reaching the OW. **b** The COMSOL Multiphysics simulation of the flow mixing on the uCR-Chip. Rapid mixing is shown by the color gradient in the mixing channel followed by uniform color of the diluted concentration in the remaining reaction channel. Controlled mixing at 1:5 ratio is illustrated in the zoom-in figure. **c** Experimental testing of the mixing on the uCR-Chip using food color dyes. The rapid mixing is shown by quick transition to the green color. Controlled mixing at the 1:5 ratio is illustrated in the zoom-in figure. **d** Illustration of a USB microscope-based colorimetric signal reading system

The uCR-Chip design (Fig. 1a) includes two inlet wells (one for the Jaffe assay reagent and the other for the sample, respectively); inlet channels with their length designed to match the desired 1:5 sample-to-reagent mixing ratio (i.e., 5L for the sample inlet channel and L for the reagent inlet channel) merging at the T-shaped mixing junction; a mixing channel to allow complete passive flow mixing, directly followed by the reaction channel for Jaffe reaction over a defined period of time under the specific flow rate; a lenticular shape observation window (OW) with a flow diverter for colorimetric signal detection; and a waste outlet well.

COMSOL Multiphysics was applied to model fluidic mixing under a constant hydrostatic pressure difference between the inlets and outlet in the uCR-Chip (Fig. 1b). The simulation results verified the expected 1:5 sample-to-reagent mixing ratio, as visualized by the ratiometric division of the mixing channel width into the dark blue and red color flows at the junction (*zoom-in image of the upper panel*, Fig. 1b). Complete mixing is demonstrated by the homogeneous blue color toward the end of the mixing channel, which remained stable over the subsequent reaction channel, the OW, and the post-OW channel to the outlet, as shown by both color visualization (*upper panel*, Fig. 1b) and the quantitative concentration plot (*bottom panel*, Fig. 1b). The normalized final concentration of 0.167 upon complete mixing, as predicted by the simulation, consistently confirmed the 1:5 sample-to-reagent mixing ratio (*bottom panel*, Fig. 1b). The same concept was experimentally demonstrated using food color dyes (Fig. 1c).

Because of the laminar flow, turbulence is effectively eliminated, and the dominant mechanism of mixing in the chip is diffusion. The relatively high diffusion coefficients of small creatinine molecules and the Jaffe reagent result in their rapid, complete mixing within just a few millimeters in the mixing channel passing the T-junction (Fig. 1b, c). Upon complete mixing, the ~62 mm long reaction channel allows the mixture to significantly react for ~4 min at the linear flow speed of ~0.25 mm/s before the reacted mixture reaches the OW.

Because the mixing ratio between two laminar flows relies solely on their relative flow rates in the inlet channels, it is critical to maintain identical pressure in the sample and reagent inlet wells. Therefore, we applied our previously established 2-PPC technique for pressure balancing^20^. Specifically, this is achieved by covering the sample and the reagent in aqueous phase in the inlet wells with a single shared volume of silicone oil, so that any pressure difference due to solution height variation in the wells is compensated by the immiscible oil phase coverage with similar mass density to the aqueous solution (Video S1).

The color signal from the uCR-Chip is imaged and analyzed by an in-house developed imaging platform (Fig. 1d). The platform was configured with a white LED backlight panel, a chip-holding stage, and a USB microscope with a bandpass blue filter (wavelength peak at 490 nm) to record the colorimetric signal at the target optical absorbance wavelength from the OW in the uCR-Chip. The captured images were then transferred to a laptop computer for analysis. Together, the standalone passive microfluidic device with accurate mixing and reaction controls and the portable, low-cost imager provides a complete PoC creatinine test prototype.

### Dry film photolithography for accurate microfluidic chip fabrication

**Fig. 2:**
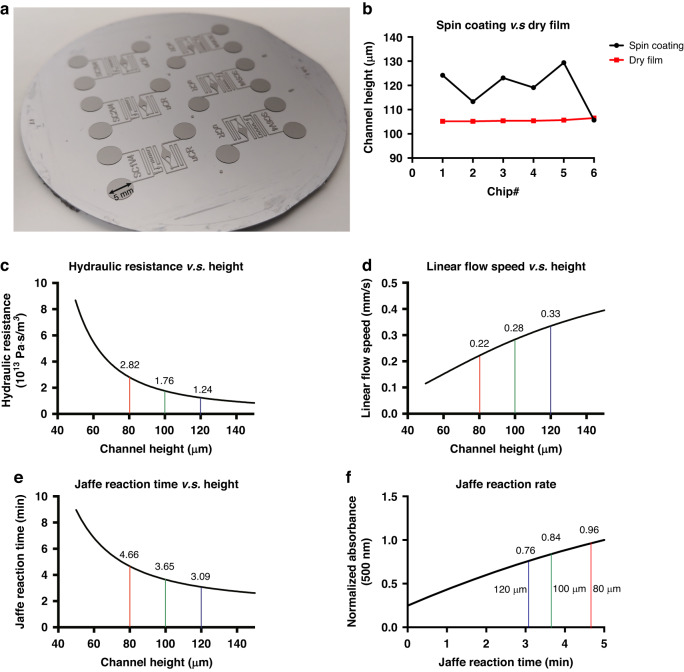
. **Comparison of the uCR-Chip fabricated by spin-coating and dry film photolithography**. **a** Photograph of the chip master fabricated by dry film photolithography on a silicon wafer, which includes six identical units; **b** Comparison of the patterned photoresist thickness fabricated with dry film or SU-8 spin coating for 6 different chips. The spin coated SU-8 2075 results in considerable pattern thickness variation (or equivalently channel height variation) among different chips. The dry film fabrication yields near identical channel height among different chips. **c**–**f** Demonstration of the effect of channel height variation on Jaffe reaction in a 60 mm long and 100 µm wide passive flow microfluidic channel. For example, the spin-coating of SU-8 2075 can lead to up to a 20 µm channel height deviation, which translates to approximately a 30–60% variation in hydraulic resistance relative the desired 100 µm channel height (**c**). Subsequently, the hydraulic resistance variation could lead to variation of linear flow speed (**d**), which ultimately affects the reaction time (**e**) before the mixture enters the OW and therefore variation of the reaction signal in the OW (**f**). The reaction time *t* is mapped to the time-dependent reaction signal *S* up to 8 min from the 20 mM creatinine sample in the well-plate assay (Fig. 4d) (nonlinear fitting function: *S* = −0.00669*t*^2^ + 0.1529*t* + 0.1972; *R*^*2*^ = 0.9982). The signal is normalized to the 8 min signal

Due to the absence of active external control, the functionality and performance of passive microfluidic systems highly depend on the specific geometric properties of the channels, especially for time-sensitive chemical reactions (Fig. 2a). Therefore, the accuracy and repeatability of the device fabrication are critical. SU-8 is a commonly used liquid photoresist for microfluidic device master mold fabrication^23–25^. Its various series of products can pattern a range of thicknesses on the substrate depending on the photoresist properties (e.g., viscosity) and the spin coating parameters. However, for more viscous SU-8 required to fabricate relatively thick patterns (e.g., SU-8 2075), spin-coating often results in considerable variation of pattern thickness. During the test we found that spin coating SU-8 resulted in up to ±20 µm variation while the dry film photoresist resulted in only <1 µm variation (Fig. 2b). Consequently, the variation in channel thickness leads to a cascade of changes in hydraulic resistance, linear flow speed, reaction time and reaction signal (Fig. 2c–f). Therefore, we chose a dry film photoresist of pre-defined thickness for master mold fabrication. A more detailed analysis of the effect of channel thickness variation on uCR-Chip is available in the Supplementary Information (Table S1).

### Optimization of signal observation window geometry

**Fig. 3:**
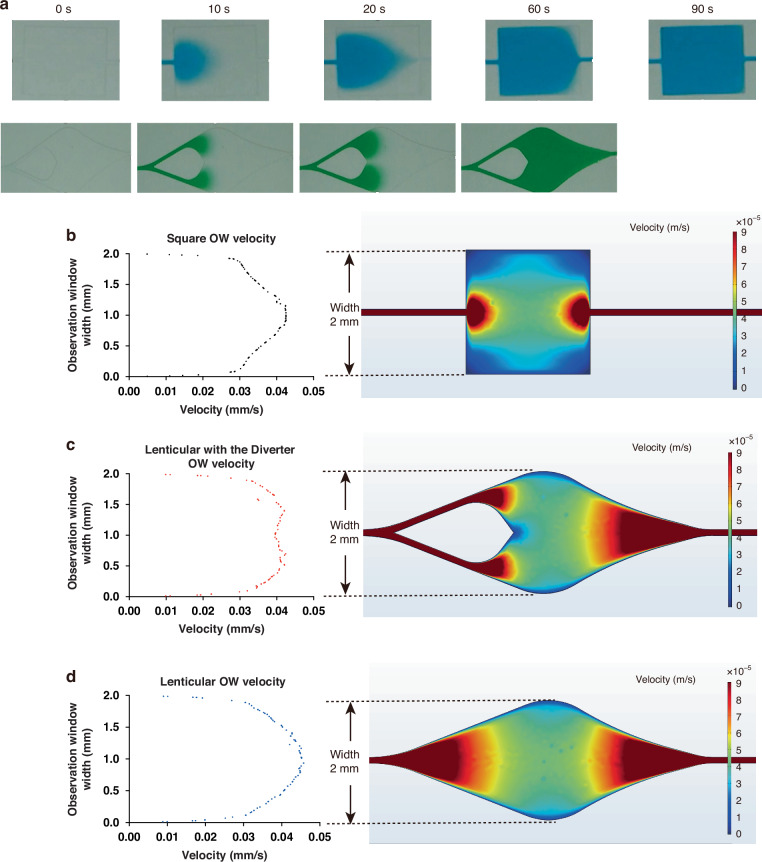
. **Optimization of signal observation window geometry**. **a** Effect of the OW geometry on OW filling time. The filling time of a typical square-shaped OW is around 90 s, while the lenticular with diverter OW geometry allows for <60 s filling time; As the food dye flows into the OW, the pre-filled water generates resistance as it is being displaced, which produces a diffusion front allowing observation of the OW filling dynamics; **b** COMSOL simulation of the flow velocity profile in the uCR-Chip with a square OW shape; COMSOL simulation of the flow velocity profile in the uCR-Chip that has a lenticular OW with (**c**) or without (**d**) a diverter. The lenticular OW with a diverter shows a more uniform flow velocity in the center region of the OW comparing to the square OW or the lenticular OW without a diverter

To achieve effective absorbance-based colorimetric signal detection in the uCR-Chip using a portable imaging platform, the OW must be sufficiently large (e.g., millimeter scale) to ensure enough pixels for accurate color measurement. However, a larger OW with a simple geometry (e.g., square shape) will require a longer filling time for the reacting mixture and is likely to create a nonuniform flow velocity profile, which complicates the distribution of the absorbance signal due to its dependence on reaction time. In this context, we hypothesized that optimizing the OW geometry could be one of the strategies to achieve a shorter OW filling time and a more uniform flow velocity profile in the OW. We designed, simulated, and tested different OW geometries (e.g., simple square, lenticular with or without a flow diverter, etc.), which helped us to select a more suitable OW design. Among these OW designs, we found the lenticular shape OW with a flow diverter optimizes the flow filling time and velocity profile. For example, it takes ~60 s to fill the lenticular + diverter OW by passive flows in the uCR-Chip compared to ~90 s to fill the simple square shape OW of similar size (Fig. 3a**;** Video S2). Furthermore, COMSOL modeling shows that a lenticular + diverter OW produces a more uniform flow velocity across the width of the OW perpendicular to the flow direction (Fig. 3b–d). We reasoned that the lenticular OW design produces a more gradual flow transition from the narrow reaction channel to the wider OW, which helps stabilize the flow. The inclusion of a flow diverting pillar at the entrance further enhances the uniformity by directing the flow closer to the OW walls and away from the center, compensating for the parabolic flow speed profile in a simple straight microfluidic channel. Thus, the lenticular + diverter OW design was chosen for the subsequent technical characterizations and validation of the uCR-Chip.

### Technical characterizations of uCR-Chip

**Fig. 4:**
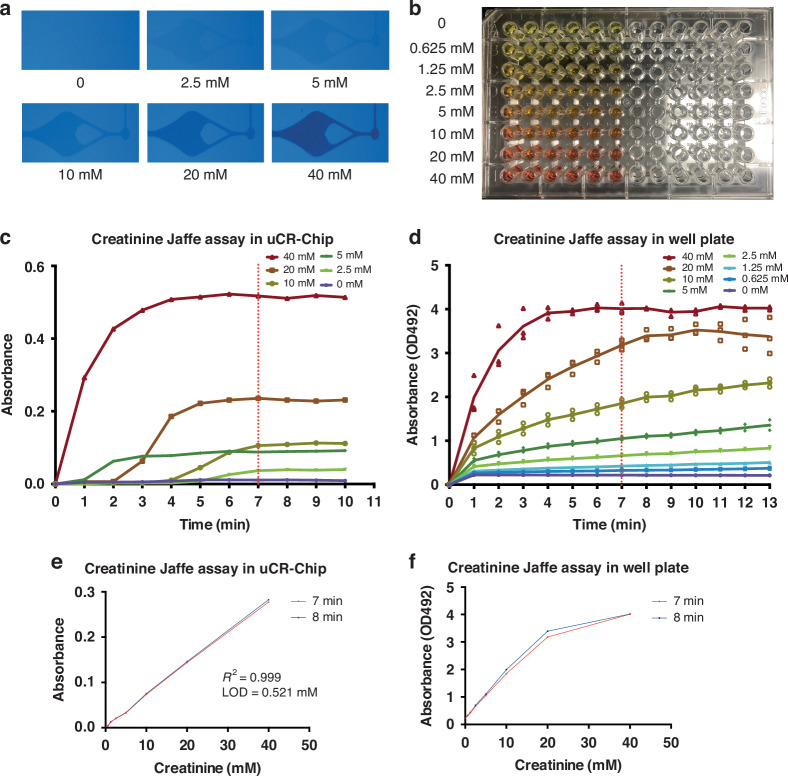
. **Technical characterizations of the uCR-Chip**. The colorimetric responses of the Jaffe reaction of different concentrations of creatinine standard up to 40 mM spiked in simple buffer as visualized in the uCR-Chip (**a**) or the well plate (**b**); **c** The Jaffe reaction signal stabilizes around 7-min for different creatinine concentrations in the uCR-Chip; **d** By comparison, the signal does not stabilize for most creatinine concentrations in the well plate assay; Calibration curve for the uCR-Chip test (based on a separate set of experiments) shows stable linear responses up to 40 mM creatinine with a LOD of 0.521 mM (**e**) in comparison to the narrower linear range and unstable calibration curve in the well plate assay (**f**)

To characterize the technical performance of the uCR-Chip, we followed our established test protocol to measure the colorimetric responses of the Jaffe assay for a range of creatinine standard concentrations in a simple buffer up to 40 mM (Fig. 4a). For each creatinine concentration, we captured time series data of the detection signal from the uCR-Chip. Our results showed that the signal increases over time and reaches a plateau within 7 min after loading the sample, reagent and applying oil coverage (Fig. 4c). The plateau mainly results from the hydrodynamic equilibrium of flows in the channels and the OW, and the level of the plateau is proportional to the creatinine concentration as expected (Fig. 4c). The 7 min signal stabilization reflects the time for mixing, reaction, OW filling, and flow stabilization. After 7 min, both the uniform color signal in the OW and the color gradient along the reaction channel remain stable. We showed the signal stability duration up to 3 min in the plot (Fig. 4c) and >30 min in the complete video recording (Video. S3). This signal stability offers a unique practical advantage for time-insensitive signal readout after 7 min of the assay in the uCR-Chip, which is particularly useful for PoC testing by the end users. Based on the time-series characterizations and signal stability results, we next repeated the tests and produced the calibration curve using the signal measurements at 7 min and 8 min (Fig. 4e). The results demonstrated a linear detection range up to 40 mM and a lower limit-of-detection (LOD) of 0.521 mM (Fig. 4e), which covers the entire clinical urine creatinine concentration range. Identical calibration curves at the two time points consistently confirmed signal stability (Fig. 4e). As a reference method, we performed the same Jaffe assay using the standard 96-well plate and a commercial plate reader for absorbance measurement (Fig. 4b). Compared to the uCR-Chip, although the well-plate assay produced a clinically applicable calibration curve (Fig. 4f), the detection signal could not stabilize even at 13 min (Fig. 4d), consistent with previous reports from the literature^21,26^. At 7 min, the linear detection range of the well-plate assay is limited to 20 mM and the LOD > 0.6 mM, and the calibration curve at 8 min considerably deviates from the 7 min calibration curve; this trend would be expected to persist over a longer time period (Fig. 4f). Collectively, the uCR-Chip exhibited superior technical performance compared to the conventional test method and met the clinical detection range and sensitivity requirements for clinical use.

### Technical validation of uCR-Chip using artificial urine

**Fig. 5:**
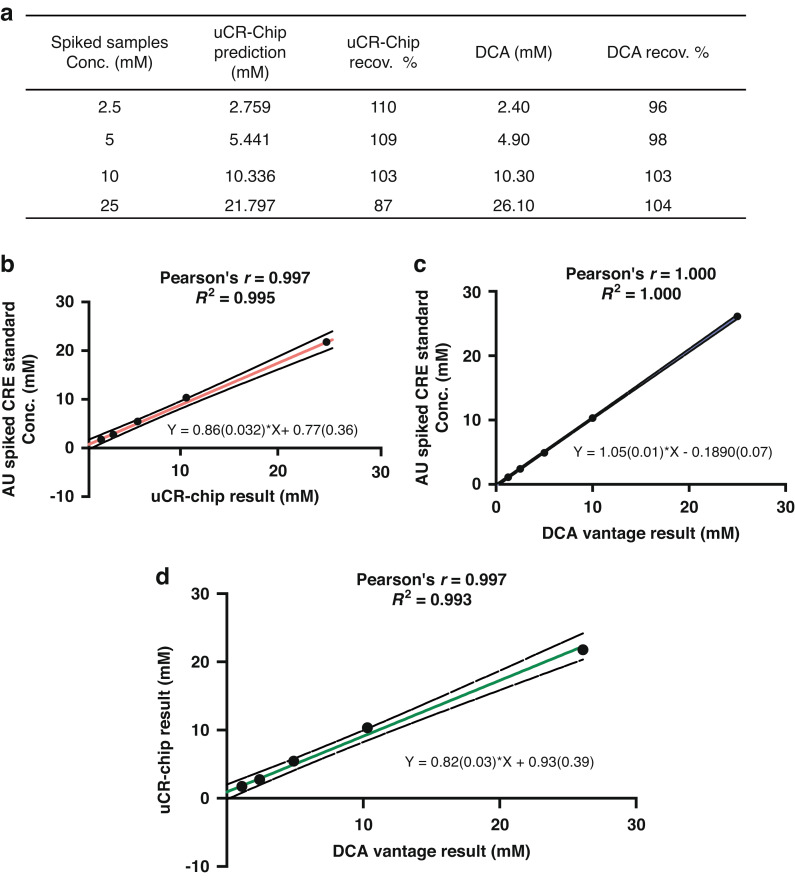
. **Technical validation of the uCR-Chip using artificial urine**. **a** The summary table of the theoretical creatinine standard concentrations spiked in artificial urine, their predictions by the uCR-Chip and the DCA Vantage test, and their respective percentage recovery. **b** The correlation plot between the uCR-Chip test prediction and the theoretical creatinine concentrations; **c** The correlation plot between the DCA Vantage test prediction and the theoretical creatinine concentrations; **d** The correlation plot between the DCA Vantage test prediction and the uCR-Chip test prediction. Pearson’s *r*, *R*^*2*^ and the linear fit functions (the number inside the bracket is the standard error of the respective fitting parameter) are shown for **b**–**d**

Based on the successful technical characterizations of the uCR-Chip, it is necessary to validate its effective use for testing urine samples. Technical validation of the uCR-Chip was performed using artificial urine for simplicity, better experimental control and to mimic the physiological matrix. The preliminary clinical sample tests data is available in Supplementary Information (Fig. S3). Specifically, creatinine standards of different concentrations (i.e., 2.5, 5, 10, and 25 mM) were spiked into commercial artificial urine. The spiked samples were then tested in the uCR-Chip, and the full uCR-Chip calibration curve was used to predict the creatinine concentration in each sample. For comparison, the same batch of spiked samples were measured using a commercial benchtop PoC urine creatinine test (i.e., the Siemens DCA Vantage^®^ test). The creatinine concentrations predicted by the uCR-Chip and the DCA test were compared with the theoretical concentrations of the spiked creatinine to evaluate recovery rate. Our results showed the expected linear correlation of the uCR-Chip predictions with the theoretical concentrations under the commonly accepted recovery rate range (i.e., 87–110% in our data) (Fig. 5a, b). Furthermore, the uCR-Chip showed a negligible base signal for the blank artificial urine sample. By contrast, the DCA test predicted a significant base creatinine concentration (4.4 mM) in the blank artificial urine control sample, suggesting its sensitivity to the matrix effect. However, upon calibration by deducting the base creatinine concentration, the DCA test predictions were more closely correlated with the theoretical concentrations and yielded a higher recovery rate (i.e., 96–104%) (Fig. 5a, c). Consistently, the predictions of the spiked creatinine concentrations by the uCR-Chip and the DCA test are correlated (Fig. 5d). Thus, the uCR-Chip was successfully validated using the artificial urine and demonstrated comparable to or even better performance than the commercial PoC test.

## Discussion

Although microfluidic devices have been increasingly developed for renal disease diagnostic applications^16^, they are not yet used in current commercial PoC urine creatinine tests. Urine dipstick tests such as the Siemens Multistix^®^ are easy to use and inexpensive, but are limited to qualitative or semi-quantitative test results and suffer from poor sensitivity and low accuracy. In comparison, sophisticated benchtop test systems such as the Abbot Afinion^TM^ and the Siemens DCA Vantage^®^ tests produce more reliable and quantitative analytical results. However, these tests require bulky, expensive, and complicated analyzers, making them non-ideal for mobile clinics, resource-limited and remote regions, or personal use. In this context, the microfluidics-based uCR-Chip balances the pros and cons of the existing tests and demonstrates its promise to enable practical urine creatinine measurement at PoC (Table S2). On one hand, the uCR-Chip is very easy to operate, only requiring simple loading steps by the users (Video S1); has a low chip material cost of under $1 US dollar per test; and the detection signal can be conveniently read by a portable and lightweight imager assembled using affordable off-the-shelf parts. On the other hand, the low-cost uCR-Chip still provides quantitative test results with detection sensitivity, range, and speed comparable to or even better than the commercial benchtop systems and offers the unique advantage of signal stability (Video S3; Table S2). Other types of microfluidic tools for biomedical detection include but are not limited to paper-based and centrifugal microfluidic platforms. For example, paper-based chips have emerged as a useful platform for rapid, easy-to-use, high-throughput and low-cost biochemical detection^27,28^. However, these devices are often limited by test accuracy, so they are primarily used as qualitative or semi-quantitative screening tools. On the other hand, centrifugal microfluidics utilizes centrifugal force to enable flexible fluid manipulation for rapid sample isolation and automated biochemical reactions^29^. However, this class of microfluidic devices is not well-suited for applications requiring continuous flow mixing, and it typically needs a special external spinning instrument, which is not ideal for PoC diagnostic applications outside research labs. In contrast, the passive-flow microfluidics approach used in the uCR-Chip enables true quantitative and accurate diagnostic testing while remaining relatively easy-to-use features due to its standalone operation. Much of the innovation in this work focuses on developing methodologies to reduce the operational skill requirements while maintaining test accuracy.

Traditionally, miniaturized microfluidic devices are powered and operated by specialized external equipment such as pumps and valves^30–33^. Various on-chip actuation strategies, such as elastic membrane valves and pumps^34–37^ or electrode arrays^38,39^, enable more flexible active fluid manipulations and create large scale, integrated, and programmable microfluidic circuits^40,41^. However, these devices still require external power or pressure sources as well as an electronic control system^42–45^, limiting them to laboratory research. For practical PoC diagnostic applications, unless the entire microfluidic system, including both the chip and the active control system, can be miniaturized, passive microfluidic devices offer some clear advantages derived from their intrinsic features of being standalone, self-operated, and external equipment-free^46–49^. The common drawback of the passive microfluidic approach is its high susceptibility to variations in device parameters and experimental conditions due to a lack of active controls, which results in accuracy and reliability concerns. In this context, the design and operation strategies used in the uCR-Chip test, including (1) accurate microfluidic channel fabrication by dry film lithography, (2) robust mixing and reaction control by the combination of the oil-coverage-based 2-PPC technique and microfluidic channel network design, (3) flexible detection signal readout time enabled by continuous-flow-based fast signal stabilization without requiring reaction equilibrium, and (4) rapid iterative device design prototyping and optimization, provide some useful reference guidelines to develop passive microfluidic devices, especially for end-user-oriented PoC diagnostic applications.

The viscosity of the reagents is a crucial factor to consider when designing a passive microfluidic system. More specifically, both the flow rate and diffusion properties of the fluid depend on viscosity, which affects fluid transport and mixing. However, in the case of the uCR-Chip, all the solutions involved in the test, including the Jaffe reagent, the creatinine standard, the artificial urine, and real urine samples, are highly diluted fluids; therefore, their viscosity is close to that of water. Consistent with this assumption, we did not observe different flow rates for these solutions in the same microfluidic channel under the same hydrostatic pressure difference between the inlet and outlet. Thus, the differential flow rate-based mixing ratio control and the diffusion-dominated fluid mixing in the uCR-Chip can be readily modeled using known physics under the assumption of viscosity identical to that of water for all solutions. However, if the sample appears to be more viscous, which is possible for some patients’ urine samples, proper dilution will be required before testing so the differential viscosity effect on flow and mixing can be minimized.

The native urine color can potentially present a spectral interference with the Jaffe reaction signal. In this regard, here we discuss our relevant considerations and some preliminary research efforts to address this issue. *First*, in our technical validation studies, we spiked creatinine standard in artificial urine, which mimics the real urine properties including its native color. Our results showed acceptable recovery rate (Fig. 5a), suggesting the urine color itself is not significantly interfering with the Jaffe reaction color signal in the uCR-Chip; *Second*, which is also related to the first, the urine sample is mixed with Jaffe reagent at the 1:5 ratio so the urine is already considerably diluted which helps reduce its native color spectral interference. To further reduce this spectral interface, the urine sample can be pre-diluted in buffer before adding to the uCR-Chip for mixing and reaction with the Jaffe reagent. However, this dilution strategy will require higher detection sensitivity of the uCR-Chip and we discussed some possible approaches to achieve it in the “Discussion” section; *Third and finally*, we have developed a new proof-of-concept prototype of the uCR-Chip that allows measurements of the Jaffe reaction kinetics through multiple serially located observation windows (OW) along the reaction channels (we called it the “kinetic uCR-Chip” or “K_uCR-Chip” due to its kinetics measurement feature or the “uCR-Abacus Chip” based on the similarity of the chip design’s graphical look to an abacus). In this new chip, the Jaffe reaction color signal over time can be measured over the OW series (Fig. S3). Thus, the relative color signal change is measured instead of the absolute color signal, which effectively eliminates the background urine color spectra interference. In similar consideration, the uCR-Chip can be modified to include an initial reference OW along the Jaffe reagent inlet channel for background color subtraction.

Previously, we successfully developed a passive microfluidic device for rapid measurement of urinary albumin (uAL-Chip)^20^. The uAL-Chip development established the core chip design and operation principles for our passive microfluidic devices. It utilizes the Albumin Blue probe to produce highly sensitive and specific fluorescent signal upon reacting with urine albumin, covering the full clinically relevant concentration range for quantitative categorization of albuminuria. However, albuminuria classification by urine albumin alone is inaccurate, requiring calibration by urine creatinine to determine uACR^50,51^. Thus, it is desirable to design a similar passive microfluidic device for urine creatinine measurement that can be readily integrated with the uAL-Chip. A practical challenge of this approach is the lack of a reliable one-step reacting fluorescent probe for detecting creatinine. As such, in the current uCR-Chip development, we chose the traditional Jaffe reaction for colorimetric quantitation of urine creatinine as a proof-of-concept bearing its known limitations^21,22^. Our envisioned dual urine markers chip will be designed to have a common sample loading inlet, which will be split and directed to interface with the uAL-Chip and uCR-Chip units for albumin and creatinine measurement, respectively, all on a single device. The 2-PPC technique can be consistently applied to the chip by covering all inlets with a common drop of silicone oil. Thus, an integrated solution for uACR determination based on passive microfluidic device can be realized (uACR-Chip). To detect signals from the uACR-Chip, a dual module portable imager capable of reading fluorescent and colorimetric signals is required for the chip’s practical use. The preliminary uACR-Chip prototype design and testing are provided in the Supplementary Information (Fig. S2). On the other hand, incorporating multi-stage mixing into the uCR-Chip will permit the more advanced enzymatic creatinine assay. The enzymatic method can be compatible with fluorescent detection signal readout and improves detection specificity, which is one of the known issues of the Jaffe assay due to positive interference with Jaffe-like chromogens. Such a multi-stage mixing approach would also facilitate the use of any other additives required for on-chip mixing control, such as the nonionic surfactant Tween-20 in the current uCR-Chip test protocol.

Although the uCR-Chip has been successfully used to test creatinine standards spiked in artificial urine, validation with real urine samples from CKD patients is expected to further validate its clinical usability. To this end, we have conducted preliminary clinical sample tests, and the data are available in the Supplementary Information (Fig. S4). Moving forward, to further clinical use of the device and automate the test, on-chip reagent storage and release-on-demand functions will be required, which can be adapted from established methods such as blister packs^52,53^. Considering the specificity issue of the Jaffe assay, samples from subjects that have abnormal endogenous Jaffe-like chromogens in urine, such as liver disease patients, jaundiced patients, and others (hemoglobinuria), would be excluded from the validation test using the current version of the uCR-Chip. With proper sample dilution (off-chip or on-chip), the uCR-Chip can predict higher creatinine concentrations in the naïve urine sample beyond the chip’s current upper detection limit, although urine creatinine higher than 40 mM is clinically uncommon. On the other hand, CKD urine samples can present higher albumin concentrations and require dilution to fit the detection range of the uAL-Chip. Thus, the low LOD of the uCR-Chip allows consistent dilution of the urine sample up to two-fold without losing sensitivity. Further improving the sensitivity of the uCR-Chip would permit higher levels of sample dilution as needed and may ultimately enable detection of the much lower creatinine concentration in blood for eGFR determination^54^. In this regard, effectively increasing the optical path to enhance light absorbance without compromising sample-reagent mixing efficiency is required. Finally, beyond CKD diagnosis, it is worth pointing out the broader biomedical relevance of urine creatinine measurement, such as monitoring athlete health, pregnancy, and drug abuse, which can all benefit from a PoC test enabled by the uCR-Chip.

## Materials and methods

### Materials

The uCR-Chip design pattern was printed using a Xerox wax printer (ColorQube 8870). A TOYO-VIEW 4 × 5 camera was used to image the chip design onto the photo film. Other materials used for photomask fabrication include the photo film (Arista Ortho Litho Film 3.0–4 × 5), the photo developer (Arista Powder A&B Litho Developer), the photo film fixer (Ilford Rapid Fixer) and the washing solution (Kodak Photo-Flo 200). The SUEX dry film photoresist was purchased from MicroLaminates (Sudbury, MA, USA) and the 3-inch silicon wafer was ordered from Silicon Inc. (Plano, TX, USA). Dow SYLGARD™ 184 Silicone Elastomer Clear was purchased from Ellsworth Adhesives (Germantown, WI, USA). The DCA Vantage™ Analyzer and DCA^®^ Microalbumin/Creatinine Urine Test were purchased from Siemens Medical Solutions Diagnostics (Tarrytown, NY, USA). The Jaffe assay reagents were purchased from Randox Laboratories (County Antrim, UK) and the traceable creatinine standard was purchased from Sigma Aldrich (Oakville, ON, Canada). Tween-20 was purchased from Sigma Aldrich (Oakville, ON, Canada). Silicone oil was purchased from Alfa Aesar (Ward Hill, MA, USA). The artificial urine (S07906) was purchased from Fisher Scientific (Ottawa, ON, Canada). Materials for the portable imaging system include a USB microscope (Dino-Lite Premier AD4113T, AnMo Electronics Corporation, New Taipei City, Taiwan), a blue bandpass filter (FB490-10-Ø1”, THORLABS) and a white LED light panel (“Lumen”) from Hey Paparazzo (Ely, Cambridgeshire, England). The Microplate Photometer (Multiskan FC) was from Thermo Scientific (Mississauga, ON, Canada).

### Microfluidic chip design and modeling

The chip pattern was designed using AutoCAD (Ver. 2023). Laminar flow mixing in the uCR-Chip was modeled in COMSOL Multiphysics (Ver. 5.5). For creatinine, the diffusion coefficient was set approximately in the order of 10^-5 ^cm²/s^55^. Since the diffusion coefficient for the Jaffe reagent (i.e., picric acid and sodium hydroxide) is not directly available in literature to our best knowledge, we approximated it to be in the same order of 10^-5 ^cm²/s based on the considerations of (1) known molecular weight of creatinine (~113 g/mol), picric acid (~229 g/mol) and sodium hydroxide (~39 g/mol); (2) the general relationship between the hydrodynamic radius and molecular weight (*r* ~ molecular weight^1/3^); and (3) the general invert proportionality between the diffusion coefficient (D) and the hydrodynamic radius (D ~ 1/ r). The Reynolds number was calculated to be ~0.025 based on the simulated flow speed of ~0.25 mm/s inside the 100 µm high mixing channel, and the fluid density and dynamic viscosity of ~10^3 ^kg/m^3^ and ~10^-3^ Pa·s respectively, ensuring the nature of laminar flow mixing in the uCR-Chip.

The inlet pressure was chosen to be 50 Pa, which represents ~5 mm of water column pressure based on the reagent or sample volume added to the inlet wells and the inlet dimensions. The outlet pressure was set to zero based on the assumption of an empty well. Because the reagent/sample loading volume is much larger than the volume of the microfluidic channels and the flow rate is relatively low, the pressure difference between the inlets and outlet can be reasonably approximated to be a constant over the test period. The height of all microfluidic chip sections was set to 100 µm in the simulation. Guided by the general inverted inlet channel length ratio to achieve the mixing ratio, the exact inlet channel length was fine tuned until the desired mixing ratio was achieved. Similarly, the mixing and reaction channel length was adjusted until complete mixing and the desired reaction time were achieved, respectively. The mixing ratio was simulated by setting the chemical concentration in the left inlet channel (short 1L) to 0 and the chemical concentration in the right inlet channel (long 5L) to unity (i.e., 1 mol/m³). Therefore, a 1:5 right-to-left flow mixing ratio produces ~0.166(6) mol/m³ upon complete mixing (Fig. 1b).

### In-house photomask fabrication

The uCR-Chip photomask was fabricated in the lab using an optical reduction technique (Fig. S1)^56^. The 9X size amplified chip design was printed to solid black patterns on a piece of printing paper using a wax printer (Xerox ColorQube 8870) for high-contrast imaging. The printed chip design was posted on a whiteboard in a well-lit room. Then the image was captured on a black-and-white Arista Ortho Litho Film 3.0 using a TOYO 4×5 film camera. The chosen film has a very low ISO of ~0.5–6, high resolution and high contrast, which was further increased using the push-pull technique (www.kodak.com). Slightly under-exposing the film helps keep the transparent regions clean followed by over-developing to darken the rest of the film.

We used a 4 s exposure time and a 5 min developing time in 1 liter of developer. The developing solution was prepared following the manufacturer’s instructions (www.freestylephoto.com). Immediately after developing, the film was rinsed in a 5% acetic acid bath (which can be replaced by citric acid) for 2 min to stop the developing process. Then the film was rinsed for another 4 min in a fixer bath (Ilford Rapid Fixer). Lastly, the film was treated with a final rinse in a water bath with 2-3 drops of wetting agent (Kodak Photo-Flo 200 Solution) to minimize water marks and promote uniform drying. The developing and rinsing in stopper and fixer baths were done in a darkroom under a red safelight.

It is worth pointing out that exact timing for exposure and developing as well as amount of light are generally important in film photography to obtain high quality photos. On the other hand, preparing a high contrast photomask for photolithography does not require precise reproduction of shades of gray. Specifically, for non-grayscale photomask, the pattern on the photo film is either opaque (black) or transparent. The photo film has a logarithmic relationship between exposure time and optic density. Therefore, low sensitivity film like the one used for our photomask fabrication is not sensitive to small variations in exposure time. This low time-sensitivity is similar for film development, which over-develops the opaque regions on the film while keeping the unexposed clear regions “untouched”. In general, the level of gray (i.e., “gamma” as termed in photography) versus developing time will eventually reach a plateau or maximum density. Therefore, the exposure and development time used in our photomask fabrication was set to allow sufficient photo film overdevelopment and contrast without requiring high precision time control. In particular, the “Arista Ortho Litho 3.0” film used in our photomask fabrication is designed for high contrast images. Collectively, these features of photo films enabled effective controls of our photomask fabrication method.

### Microfluidic chip fabrication

The chip mold was patterned using a 100 µm thick SUEX dry film photoresist on a 3-inch silicon wafer following the standard dry film photolithography protocol in the Nano Systems Fabrication Laboratory at the University of Manitoba. Then the photoresist pattern was transferred to the polydimethylsiloxane (PDMS) replica using the standard soft-lithography method^57^. The PDMS replica of 5 mm thickness was cast from the mold, and 5 mm diameter holes were punched to serve as the inlet and outlet wells. A separate 2 mm thick blank PDMS slab with a rectangular shape hole that matches the size of the inlet wells of the channel-containing PDMS replica was prepared separately. The two PDMS slabs were O_2_ plasma bonded, with the rectangular hole of the blank PDMS aligned to the inlet wells of the channel-containing PDMS replica, such that oil coverage for the inlet wells is confined to a defined volume. Finally, the entire PDMS slab was air plasma bonded to a clean glass slide to seal the channels. The microfluidic channels and all the inlet and outlet wells were filled with DI H_2_O immediately after plasma bonding to maintain hydrophilicity, and the chip was kept inside a clean bench until use, typically within the same day.

### uCR-Chip test protocol

The Jaffe assay working solution was prepared according to the manufacturer’s instructions. Specifically, R1 (1% picric acid) and R2 (0.75N NaOH) were mixed at 1:1 ratio and the mixed working solution was immediately used to react with creatinine^21,22^. Tween-20 was added to the sample (creatinine standard spiked in DI H_2_O, artificial urine or real urine sample) and the working solution at the final concentration of 0.05% (V/V), which is required to prevent back flow at the mixing junction of the uCR-Chip by reducing surface tension in the current test protocol. Immediately before the test, water from inlet and outlet wells was emptied using a pipette. Then, the prepared sample and the working solution, each at ~60 μL volume, were loaded simultaneously using a multi-channel pipette. This was followed by adding a common drop of silicone oil to cover both inlets and fill the entire oil confinement reservoir, so that the total solution height in the inlet wells is consistently balanced (Video S1). An original image of the OW before loading the reagents was taken as the chip’s internal reference control (i.e., the 0th minute image) and a second image of the OW was captured after 7 min (the 7th minute image) using the USB microscope-based image platform. For the signal stability test, the images were recorded for a longer time at shorter time intervals. The acquired images were analyzed using ImageJ (Ver. 1.49 v) to measure the average OW blue channel intensities *I* and *I*_*0*_ for the 7th minute image and the 0th minute image, which were used to calculate transmittance *T* and absorbance *A* (Eq. 3). Following the Beer-Lambert law^58^, absorbance *A* is directly proportional to the molar absorption coefficient ***ε*** of the particular substance, the molar concentration *c*, and the optical path length *l*. Thus, the absorbance linearly correlates with the concentration of the Janovsky complex and equivalently correlates with the creatinine concentration in the sample.

3 $$A=-{\log }_{10}\left(T\right)=-{\log }_{10}\left(\frac{I}{{I}_{0}}\right)=\varepsilon {cl}$$

### Creatinine Jaffe assay in well-plate

The Jaffe assay was conducted in the well plate according to the manufacturer’s instructions. R1 (1% picric acid) and R2 (0.75N NaOH) were mixed at a 1:1 ratio, and the mixed working solution (25 µL R1 mixed with 25 µL R2 for each reaction) was immediately used to react with the creatinine standard sample (5 µL). Tween-20 was added to the sample (creatinine standards of 0.625, 1.25, 2.5, 5, 10, 20, and 40 mM prepared in DI H_2_O) and the working solution at a final concentration of 0.05% (V/V) to be consistent with the uCR-Chip testing. The absorbance value (O.D. at 492 nm) of each reaction in the 96-well plate was measured by a plate reader at different time points up to 13 min.

### DCA Vantage^®^ ACR test protocol

The DCA Vantage^®^ ACR test (in which the creatinine test is based on the Benedict-Behre reaction) was used as a commercial PoC creatinine test for comparison with the uCR-Chip test. The DCA test was conducted using the test cartridge from the kit and the DCA Vantage analyzer following the manufacturer’s instructions. The tip of the capillary tube was inserted into the creatinine standard sample (~100 µL per sample; four sample groups: 2.5, 5, 10, and 25 mM creatinine spiked in the commercial artificial urine) up to the level above the starch plug. The capillary holder was then secured into the cartridge until it snapped into place. Subsequently, the cartridge was inserted into the analyzer system, the plunger was depressed into the cartridge, the flexible pull-tab was removed, and the compartment door was closed to initiate the assay within the analyzer.

### Data analysis

Statistical analysis was performed using GraphPad Prism 6.0. Each individual test was repeated at least three times. The detection signal data and the creatinine concentrations were linearly interpolated to produce the calibration curves. The linearity of the regression was evaluated by the coefficient of determination *R*^*2*^. The LOD was calculated using the standard method from the linear regression.

4 $${LOD}=3\cdot \left(\frac{\sigma }{s}\right)$$

where *σ* is the standard deviation of the regression intercept and *s* is the regression slope respectively.

For the validation test using creatinine standard spiked in artificial urine, the recovery rate was calculated as (experimental prediction ÷ theoretical value) × 100%. The Pearson’s correlation coefficient *r* was applied to quantify the linear correlation between two sets of data.

## Supplementary information

Demonstration of the Jaffe reaction color signal development on the uCR-Chip

Comparison of OW filling efficiency

Demonstration of Jaffe reaction signal stability in the uCR-Chip

Supplementary information
