# Supplementary material for: A passive flow microreactor for urine creatinine test
Source: Microsyst Nanoeng. 2025 Apr 2;11:56. doi: 10.1038/s41378-025-00880-z (PMC11965425; doi:10.1038/s41378-025-00880-z)
Supplement: Supplementary file 4 — Supplementary information [file 41378_2025_880_MOESM4_ESM.docx]

**A passive flow microreactor for urine creatinine test**

Dumitru Tomsa^1#^, Yang Liu^1#^, Amanda Stefanson^1#^, Xiaoou Ren^1,2^, AbdulRazaq A. H. Sokoro^3,4^, Paul Komenda^4^, Navdeep Tangri^4^, Rene P. Zahedi^4,5^, Claudio Rigatto^4^*, Francis Lin^1^*

^1^Department of Physics and Astronomy, University of Manitoba, Winnipeg, Manitoba R3T 2N2, Canada

^2^Institute of Health Sciences and Technology, Institutes of Material Science and Information Technology, Anhui University, Hefei, 230601, China

^3^Department of Pathology, University of Manitoba, Winnipeg, Manitoba, R3P 3E5, Canada.

^4^Department of Internal Medicine, University of Manitoba, Winnipeg, Manitoba, R3A 1R9, Canada

^5^Manitoba Centre for Proteomics and Systems Biology, Winnipeg, Manitoba, R3E 3P4, Canada

^#^These authors contributed equally

*Co-senior authors: francis.lin@umanitoba.ca for F.L.; crigatto@sbgh.mb.ca for C.R.

**Supplementary Information**

**Preliminary clinical samples test with uCR-Chip**

The study protocol was approved by the University of Manitoba Human Research Ethics Board and the Seven Oaks Hospital Health Research Review Board. Under the informed consent, urine samples from CKD patients were collected at the Seven Oaks General Hospital in Winnipeg. Following the protocol in the Method section of the manuscript, we measured the creatinine level in the 10 CKD urine samples using both the uCR-Chip and the commercial DCA test (**Fig. S4a**). The clinical standard Deming regression and the conventional linear regression as part of a specialized software package (EP Evaluator) are applied for the comparison analysis, and the preliminary results showed relative comparable test data between the uCR-Chip and the DCA test (**Fig. S4b**).

**Comparison of microfluidic device fabricated by dry film photolithography vs*.* SU-8 spin-coating based photolithography for uCR-Chip test**

To demonstrate the importance of microfluidic channel fabrication accuracy for the uCR-Chip test, we provide a brief analysis of the effects caused by channel height variations. In the example discussed in this section, we consider a single microfluidic channel with a fixed length, width, and hydrostatic pressure difference between the inlet and outlet. Assuming the microfluidic reaction channel has a length and width of 62 mm and 100 µm, respectively, a 20 µm channel height variation leads to a significant variation in hydraulic resistance (**Table S1**). Considering the entire microfluidic channel network and OW of the uCR-Chip, the hydraulic resistance variation can be even more pronounced, depending on the exact design. Further, with a hydrostatic pressure difference of 50 Pa between the inlets and outlet (i.e., ~5 mm water column pressure), the hydraulic resistance variation alters the linear flow speed, which in turn affects the Jaffe reaction time before the mixture reaches the OW, assuming complete mixing at the beginning of the reaction channel.

Consequently, since the Jaffe reaction signal remains in the rising phase at the end of the reaction channel (as a longer channel would extend the test time beyond the desired rapid PoC test and is not necessary for accurate quantification), variations in reaction time lead to different absorbance signal measurements in the OW. Altogether, this simplified calculation demonstrates the importance of channel height accuracy for the uCR-Chip and supports the usefulness of dry film mold fabrication. Although not directly addressed here, dry film mold fabrication is reasonably predicted to achieve better channel thickness uniformity across the chip than spin-coating SU-8, thus improving accuracy and consistency in the reaction signal. Therefore, the use of dry film photolithography is critical to ensuring the reliability and repeatability of uCR-Chip tests.


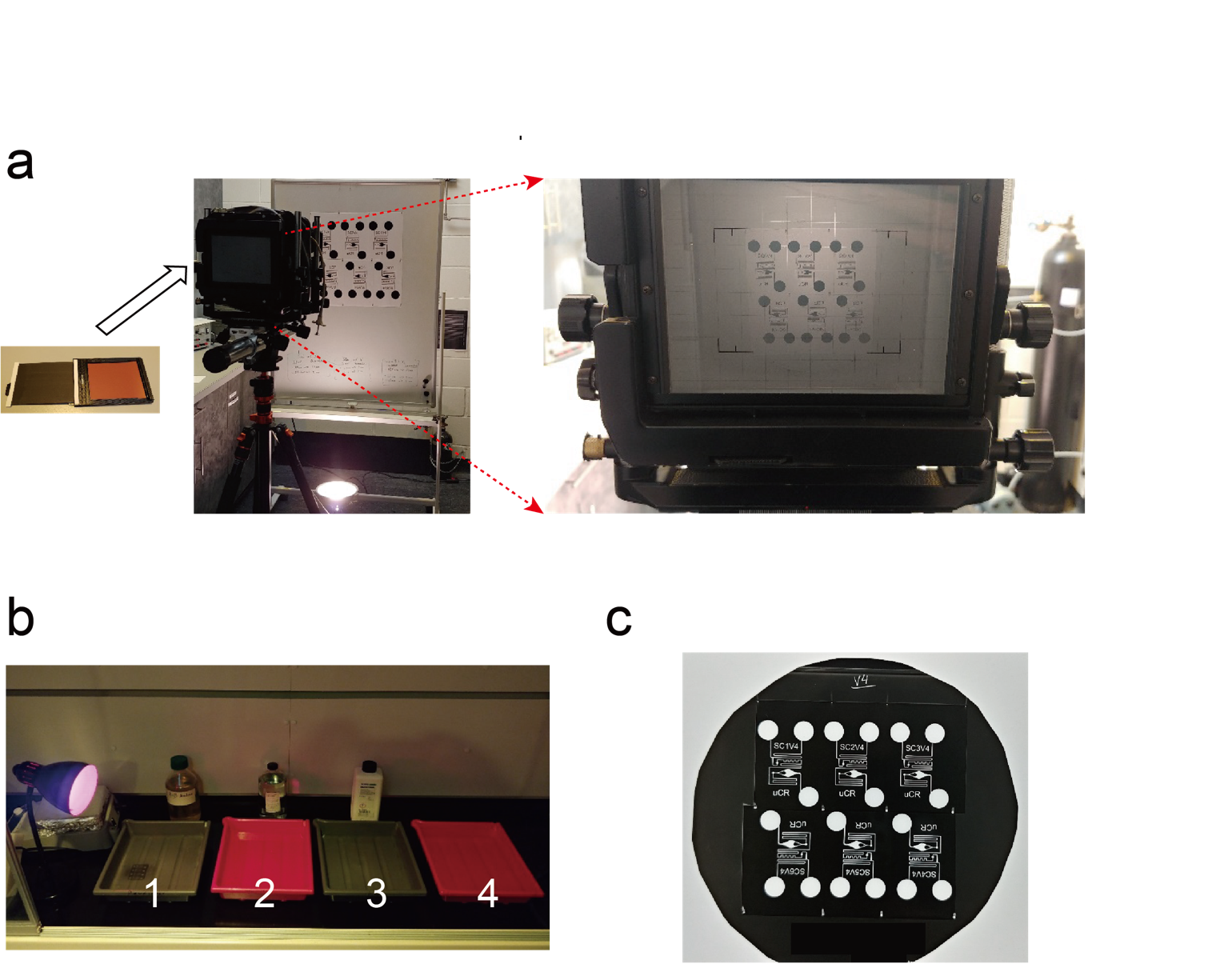


**Fig. S1. Illustration of the in-house uCR-Chip photomask fabrication method.**

**a.** **Illustration of the photomask imaging.** The design pattern of the uCR-Chip was printed onto a printing paper using a wax printer for high contrast imaging. The printout was attached on a white board using small magnets; For imaging, the film was loaded to the film frame, which was then installed to the camera. The distance from the camera to the board depends on the optics and the reduction scale of the printed design pattern to the photomask film. In our case, the distance is ~140 cm – 180 cm (~55” – 70”) and the scale is ~9:1. Once the film was exposed, the film frame cover was put back and the film frame was stored until ready for developing. **b.** **Illustration of the film developing steps.** The film developing was done in 4 trays in sequence. Tray 1 is filled with the developer (1:1 mix of parts A and B). The developing time is 5.5 min. Tray 2 is filled with vinegar (stopper) that stops the action of the developer. Two minutes in the stopper is generally enough to stop the reaction. Tray 3 is filled with the fixer solution to fix the developed pattern on the film. Four minutes of soaking is typically sufficient. Tray 4 is filled with water with a drop of surfactant (Kodak Photo-Flo). This step makes sure that the film is clean and dries without stains. **c.** **Picture of a finished uCR-Chip photomask.**

**
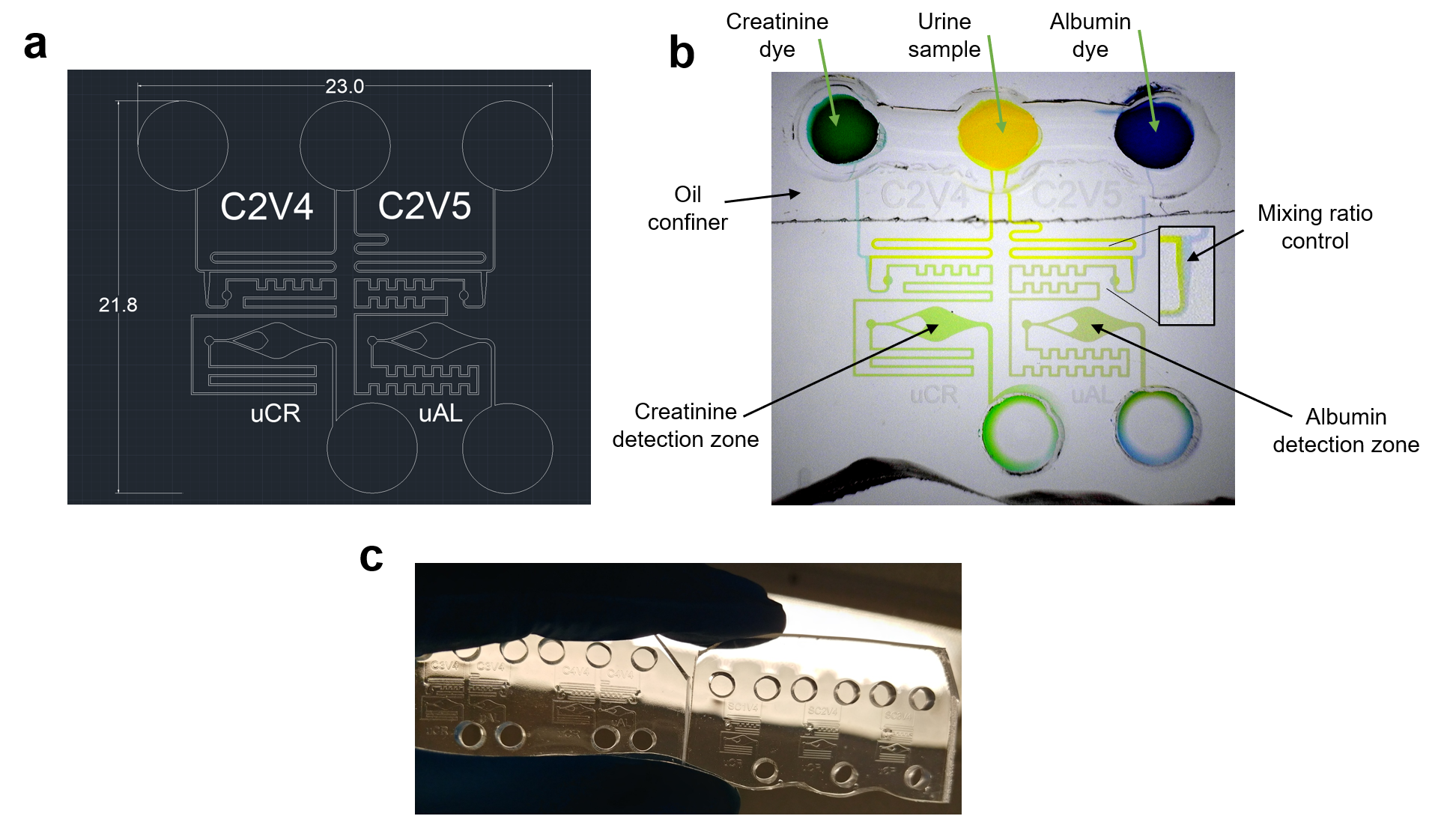
**

**Figure S2. Demonstration of the dual urine albumin and creatinine test chip.**

**a.** **The schematic design of the integrated dual urine markers test chip.** Dimension labels are in millimeters. **b. Demonstration of the integrated chip test using food color dyes to mimic mixing and reaction.** **c. Example of the integrated dual makers test chip (left; 2 unites per chip) and the single marker test chip (right; 3 units per chip).** Labels of different combination of letters and numbers are for practical labeling of the chip designs only.

*
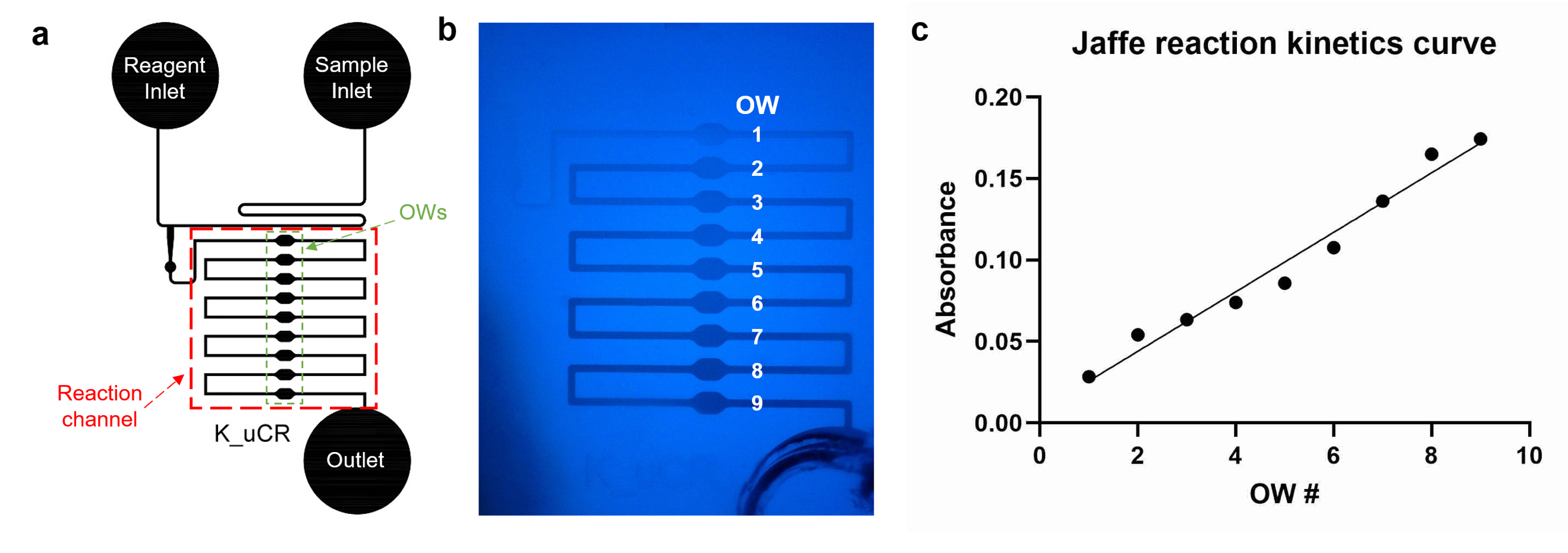
*

**Figure S3. uCR-Abacus Chip for measuring Jaffe reaction kinetics**.

**a. The schematic design of the uCR-Abacus Chip; b. Example image of the Jaffe reaction in the** **uCR-Abacus Chip, where the darkness of the color signal over the OW series is demonstrated; c. Plot of the color signal intensity vs. OW number of the uCR-Abacus Chip in b).**

**Figure S4. Preliminary validation of the uCR-Chip test with 10 clinical urine samples from CKD patients.**

**a. Comparison of the uCR-Chip test data with the commercial DCA creatinine test data of the 10 CKD urine samples**. **b. The clinical standard Deming regression and conventional linear regression are applied to compare the uCR-Chip test and the DCA test of the 10 CKD urine samples.** The blue line shows linear regression and the pink line shows Deming regression (R^2^ = 0.84). The black dashed lines indicate the 95% confidence intervals for the linear regression. In addition, the data was analyzed by a specialized software EP Evaluator (which consistently applies the Deming regression and linear regression for comparison analysis) and the result shows that the uCR-Chip is considered an alternative quantitative method to the DCA method. Furthermore, the paired *t*-test gives the *p* value of 0.17, indicating no statistically significance difference between the uCR-Chip test and the DCA test.


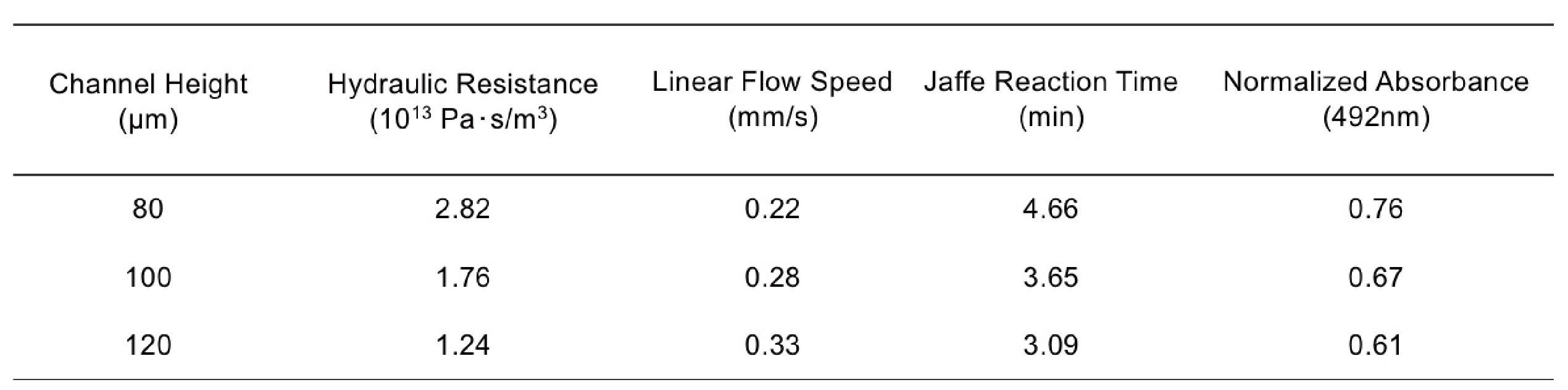


**Table S1: Example comparison table of hydraulic resistance, linear flow speed, reaction time and reaction signal for three different channel height (i.e. 80 µm, 100 µm and 120 µm)**

**Table S2: Comparison of the uCR-Chip with selected commercial PoC urine creatinine tests**

**Refs**:

1. <https://emerdepot.com/products/siemens-clinitek-microalbumin-test-strip?currency=CAD&variant=43796170834144&utm_source=google&utm_medium=cpc&utm_campaign=Google%20Shopping&stkn=f360e0322162&utm_source=google&utm_medium=cpc&utm_campaign=18315354949&utm_content=&utm_term=&gad_source=1&gclid=Cj0KCQjwsaqzBhDdARIsAK2gqnfYWBBO3Xx4BHIr9qsu90TM5IGcmXl0A29FIQbVK5QFC583YDJMQvIaAsFVEALw_wcB>
2. <https://shtg.scot/media/2171/minuteful-kidney-for-home-testing-of-albumin-to-creatinine-ratio-acr-imtov10.pdf>
3. <https://stat-technologies.com/product/afinion-acr-test-cartridge/>
4. <https://stat-technologies.com/product/siemens-dca-vantage-creatnine-capillary-holders-10pk-copy/>

**Video S1: Demonstration of sample, Jaffe reagent and oil loading to the uCR-Chip, and the subsequent Jaffe reaction color signal development on the chip.**

**Video S2: Comparison of OW filling efficiency between the square shape and the lenticular shape with flow diverter**

**Video S3: Demonstration of Jaffe reaction signal stability in the uCR-Chip**
